# Computational B-cell epitope identification and production of neutralizing murine antibodies against Atroxlysin-I

**DOI:** 10.1038/s41598-018-33298-x

**Published:** 2018-10-08

**Authors:** Edgar Ernesto Gonzalez Kozlova, Loïc Cerf, Francisco Santos Schneider, Benjamin Thomas Viart, Christophe NGuyen, Bethina Trevisol Steiner, Sabrina de Almeida Lima, Franck Molina, Clara Guerra Duarte, Liza Felicori, Carlos Chávez-Olórtegui, Ricardo Andrez Machado-de-Ávila

**Affiliations:** 1 Departamento de Bioquímica e Imunologia, Instituto de Ciências Biológicas, Universidade Federal de Minas Gerais, Avenida Antônio Carlos, 6627 Belo Horizonte, Brazil; 2 Departamento de Ciências da Computação, Universidade Federal de Minas Gerais, Belo Horizonte, Brazil; 3 Sys2Diag UMR 9005 CNRS/ALCEDIAG, Complex System Modeling and Engineering for Diagnosis, Montpellier, France; 4 UMR 8030, CNRS, Université Évry-Val-d’Essonne, CEA, Institut de Génomique - Genoscope, Laboratoire d’Analyses Bioinformatiques pour la Génomique et le Métabolisme, F-91000 Évry, France; 5 Laboratório de Biologia Celular e Molecular, Programa de Pós-graduação em Ciências da Saúde, Universidade do Extremo Sul Catarinense, Criciúma, Brazil

## Abstract

Epitope identification is essential for developing effective antibodies that can detect and neutralize bioactive proteins. Computational prediction is a valuable and time-saving alternative for experimental identification. Current computational methods for epitope prediction are underused and undervalued due to their high false positive rate. In this work, we targeted common properties of linear B-cell epitopes identified in an individual protein class (metalloendopeptidases) and introduced an alternative method to reduce the false positive rate and increase accuracy, proposing to restrict predictive models to a single specific protein class. For this purpose, curated epitope sequences from metalloendopeptidases were transformed into frame-shifted Kmers (3 to 15 amino acid residues long). These Kmers were decomposed into a matrix of biochemical attributes and used to train a decision tree classifier. The resulting prediction model showed a lower false positive rate and greater area under the curve when compared to state-of-the-art methods. Our predictions were used for synthesizing peptides mimicking the predicted epitopes for immunization of mice. A predicted linear epitope that was previously undetected by an experimental immunoassay was able to induce neutralizing-antibody production in mice. Therefore, we present an improved prediction alternative and show that computationally identified epitopes can go undetected during experimental mapping.

## Introduction

Correct epitope identification is essential for developing vaccines and selecting high-affinity antibodies for immunotherapy and immunodiagnostics^1^. Experimental epitope identification is an expensive procedure and comprises several challenges. These challenges include antibody production to identify antigenic regions in a target protein, adequate animal models, and further epitope validation through crystallography. Besides, high-affinity antibody methods and immunoassays can contradict each other on which region is a better target. On the other hand, computational approaches can help to guide experimental assays and improve precision by selecting specific regions with high probability of being effective epitopes^2^.

Attempts to predict B-cell epitopes started in the 70 s^3^. They focused on amino acid properties within a sequence, such as hydrophobicity, hydrophilicity, or antigenicity, to identify propensities and patterns^4,5^. Exhaustive benchmark procedures for (and rigorous statistical analysis of) the biochemical properties that influence epitopes have revealed that single-scale amino acid profiles cannot be used to reliably predict epitope localization^6,7^. Current classification techniques involve a combination of attributes to increase the information gain. These methods are sensitive to data quality and are often subject to under- or over-representation of attributes. This approach can produce false negatives and positives, which—despite good accuracy (area under the curve [AUC] of 0.7)—lead to misidentification of epitopes^7^.

The experimental methods that accompany computational identification have limitations. Often, different wet-lab techniques are in disagreement on the important regions in a protein, thus resulting in highly heterogeneous epitope composition^8,9^. The removal of this experimental noise to train proper classifiers has been attempted by combining attributes, but the results have not been significantly better than those obtained with a few physicochemical attributes^4,10,11^.

It is generally accepted that most epitopes are conformational^12^, but even though some algorithms focus on structural properties to target these epitopes, prediction has not improved^13,14^. A key factor for achieving greater success in separating epitopes from the background is a reduction in both computational and experimental bias^8,15^. Public databases compiled from validated information^16^ and statistical analyses^17^ are essential for building adequate computational models designed for epitope prediction^18,19^. Moreover, it should be considered that immunological attributes are strongly related to animal models and evolutionary traits of a protein^20–22^.

Restricting the problem to a specific antigen group may lead to more precise epitope prediction because of the increase in information quality and a reduction in the noise from other protein groups. For testing this hypothesis, we focused on metalloendopeptidases carefully curated by means of available information to predict epitopes^16,23^. This article describes a methodology—based on a decision tree classifier—for identifying epitopes in a single protein class and for producing neutralizing antibodies against target proteins. The proteins used to experimentally validate our hypothesis belong to the venom of three snake species: *Bothrops atrox*, *Bothrops asper*, and *Bothrops leucurus*. Snakes from the *Bothrops* genus cause more than 80% of yearly snakebite accidents in Brazil^24^, thus being medically significant^25,26^. These venoms exert proteolytic action with well-known biological effects such as hemorrhage that can be evaluated *in vitro* and *in vivo*^27,28^. These characteristics allow for studying the neutralizing-antibody capabilities on the basis of our computational and experimental results, thereby, unveiling antigenic and immunogenic protein properties.

## Results

### Kmer classification rules allow for reducing false positives from nonepitope residues

**Figure 1:**
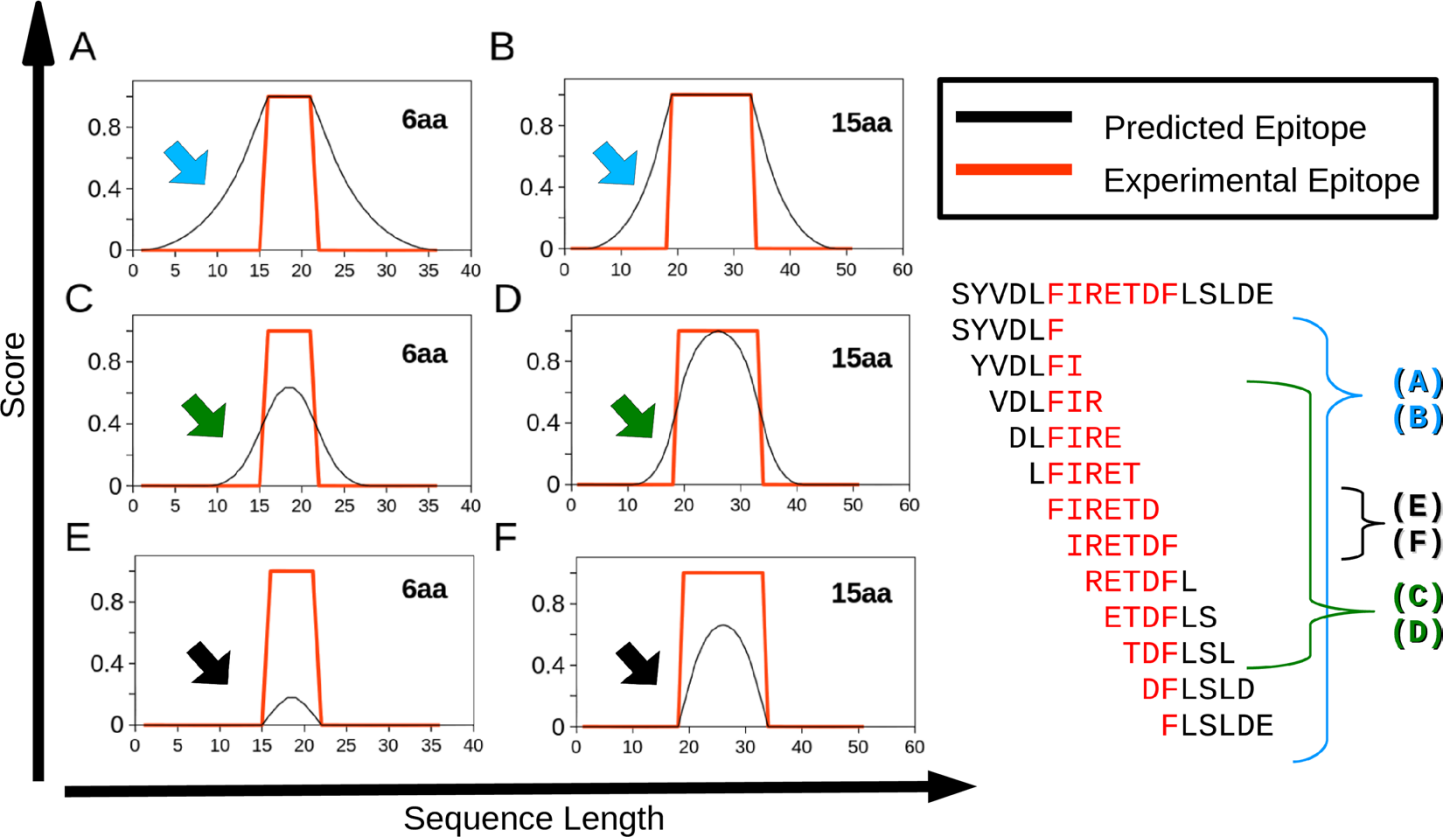
Selection of Kmers as epitopes. The graphs illustrate how the selection of positive Kmers to be considered epitopes alters the rate of false positives based on compositional rules. As an example, Kmers of 6 and 15 aa were employed. The X-axis shows the amino acid sequence position. The Y-axis shows the probability of an amino acid residue to be a part of an epitope. Red lines represent a true epitope. Black lines represent computational prediction. (**A**) and (**B**) illustrate a prediction where at least one amino acid residue must be predicted as an epitope to label a Kmer as positive (**C**) and (**D**) show that when 50% of the amino acids must be predicted as an epitope to label a Kmer as positive (**E**) and (**F**) shows when all the amino acid residues from of a Kmer must be predicted as an epitope to label a Kmer as positive. The arrows indicate the portion of potentially false positives in each prediction method. On the right side of the figure, there is an example of prediction of an epitope marked red within the sequence SYVDLFIRETDFLSLDE by means of a 6 aa Kmer and the three approaches illustrated in the graphs.

Our manually curated dataset is available in Supplementary Material 1. It contains 40 epitopes, as described in the Methods section. This dataset was transformed into a matrix summarizing 101,115 elements representing Kmers of 3 to 15 amino acid residues (aa), each described by 33 attributes. When a Kmer maps 50% or more of its length onto an epitope or nonepitope, it is assigned to that respective class by our approach. This method produced fewer false positives than did other labeling methods, as illustrated in Fig. 1. Red lines indicate a true epitope, while black lines represent computational prediction. The example shows Kmers of 6 and 15 aa under three selection conditions: when only 1 aa is required to label the Kmers as an epitope (A and B), when the 50% or more rule used in our method is applied (C and D), and when the exact epitope must be matched to regard a Kmer as an epitope (E and F). It must be highlighted that the method proposed here is less sensitive for small epitopes (under 5 aa), but it can be later corrected by the SMOTE algorithm during the decision tree classifier training.

### Charged amino acid residues contribute to B-cell epitope prediction with a decision tree model

We used a decision tree classifier for predicting epitopes of three metalloendopeptidases from *Bothrops* snake venoms (Bap1, Atr-I, and Leuc-a). Epitopes derived from these proteins were not present in our classifier training dataset. The classification tree is presented in Supplementary material 2. A comparison with random-forest attribute analysis highlighted the importance (for the aliphatic index) of the percentages of Arg (R), His (H), Lys (K), Glu (E), Asp (N), Pro (P), and Trp (W). Table 1 summarizes this comparison. The highlighted attributes represent the first nodes of the decision tree model. Decreased Gini values are an inequity measure between epitope and nonepitope classes. The lower values represent the best attributes across a million trees growth with random forest.

**Table 1:** Valuable attributes from random-forest and decision tree classifiers.

Attribute	Decreased Gini	Decreased Accuracy
Positive charged RHK	75,28	37.50*
Negative charged DE	79,12	52,71
Uncharged STNQ	101,78	63,27
Special CGP	100,9	43.43*
Hydrophobic AVILMFW	92,84	43.16*
gravy	198,59	60,82
**Aliphatic index (5)**	118,75	45.49*
% Atoms of C	234,22	76,8
% Atoms of H	212,17	58,34
% Atoms of N	233,88	59,07
**% Atoms of O (12)**	198,23	68,39
**% Atoms of S (1)**	166,32	67,65
**% Arg (4)**	72,98	39.25*
**% His (2)**	68.85*	53,73
**% Lys (3)**	36.46*	42.84*
% Asp	80,68	56,97
**% Glu (6)**	66.12*	52,07
% Serine	107,13	76,88
**% Thr (11)**	94,8	73,62
**% Asn (9)**	74,87	47.39*
% Gln	73,67	55,23
% Cys	77,22	45.50*
% Gly	98,59	52,81
**% Pro(10)**	58.36*	39.87*
% Ala	86,08	65,12
% Val	48.48*	38.31*
% Ile	68.87*	40.67*
% Leu	74,26	42.55*
% Met	87,91	61,82
% Phe	48.65*	39.62*
% Tyr	64.44*	56,46
**% Trp(8)**	57.03*	51,84
**Isoelectric point (7)**	183,09	54,9

The attributes in bold represent the first nodes of the decision tree and (*) indicate the best attributes from random-forest.

### Experimental and computational B-cell epitope mapping

**Figure 2:**
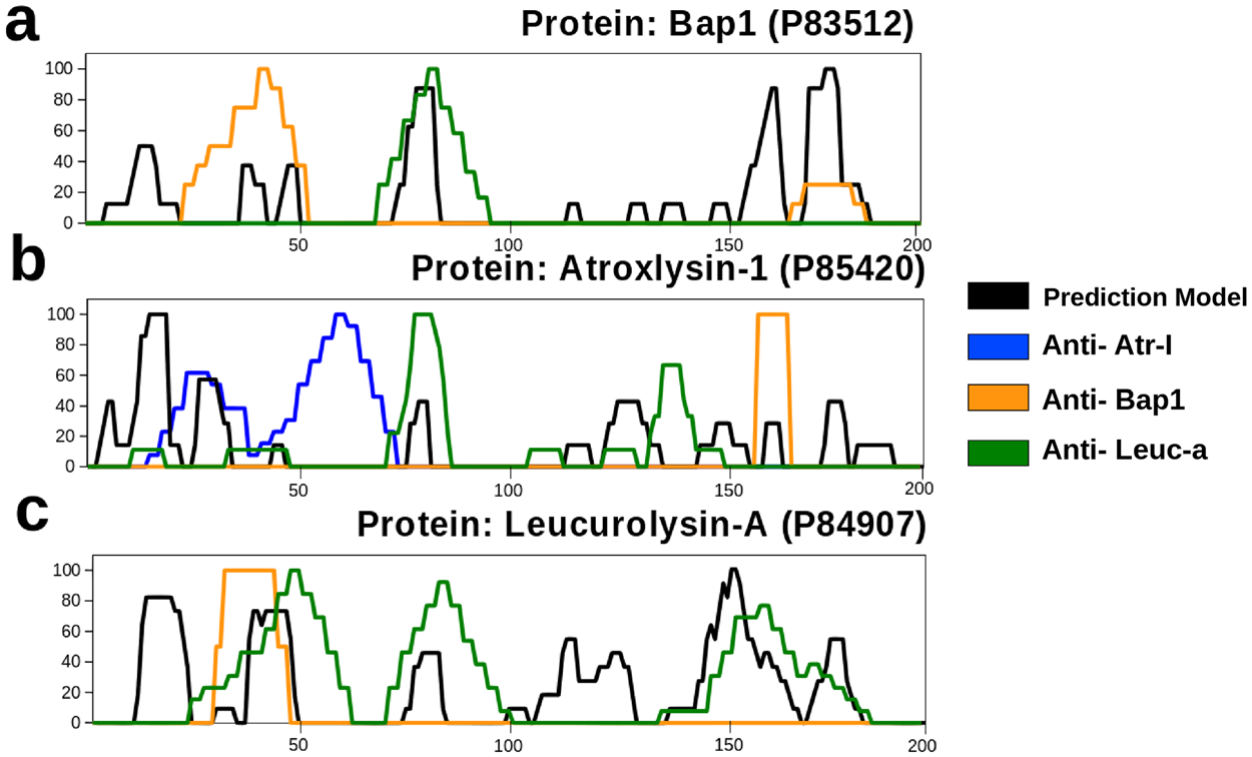
Predicted and experimental epitope overlapping. The X-axis shows amino acid residue position. The Y-axis represents the experimental and predicted epitope score values from 0 to 100. Black lines represent the epitopes predicted by our model. The blue, orange, and green lines represent epitope mapping by SPOT-Immunoblotting using anti-Atr-I, anti-Bap1, and anti-Leuc-a antibodies respectively. Letters (**a**–**c**) represent the mapping of epitopes within the individual proteins, Bap1, Atr-I, and Leuc-a from *Bothrops asper*, *B. atrox*, and *B. leucurus*, respectively. The overlapping positions between black and colored lines represent successful predictions, while overlapping between colored lines indicates a cross-reaction.

To compare our computational prediction method with experimental approaches, we used SPOT immunoblotting to map epitopes within metalloendopeptidases Atr-I, Leuc-a, and Bap1, using specific antibodies developed against each protein. Each protein was probed with all three antibodies: anti-Atr-I, anti-Leuc-a, and anti-Bap1. We identified two epitopic regions for Atr-I (aa 19–39 and aa 46–75), shown as blue lines (Fig. 2b), three regions for Leuc-a, highlighted as green lines (Fig. 2c), and two regions for Bap1, presented as orange lines (Fig. 2a). The local alignment showed that Atr-I shares a sequence identity of 55.45% and 50% with Bap1 and Leuc-a, respectively, while Leuc-a and Bap1 share 78.22% identity (Table 2). Besides, SPOT-identified epitopes presented similar position within the protein sequences.

**Table 2:** Clustal Omega Identity matrix between metalloproteinases.

	Atr-I	Leuc-a	Bap1
Atr-I	100	50.5	55.45
Leuc-a	50.5	100	78.22
Bap1	55.45	78.22	100

We predicted eight epitopic regions for Atr-I (positions 4–6, 12–20, 28–35, 79–83, 127–136, 153–156, 165–168 and 180–183), six epitopic regions for Bap1 (13–18, 39–44, 49–52, 77–86, 161–169, and 176–188), and six epitopic regions for Leuc-a (11–24, 38–48, 78–84, 114–130, 147–168, and 176–183; Table 3). These predicted regions successfully matched experimental results (Fig. 2). The experimental mapping by SPOT yielded two epitopes in Bap1 (orange lines), two in Atr-I (blue lines), and three in Leuc-a (green lines). These regions were identified by means of specific sera against each of these toxins. Cross-reaction was also observed when we employed different antibodies against each protein. The anti-Leuc-a antibody, when used against the Bap1 spot membrane, recognized a region different from that recognized by anti-Bap1 serum. On the other hand, anti-Atr-I sera did not recognize any epitope from Bap1. Furthermore, the anti-Leuc-a antibody when used against Atr-I, recognized two central regions different from those recognized by anti-Atr-I or anti-Bap1 sera. Anti-Leuc-a identified a single epitope region close to the C-terminal Atr-1 segment. Anti-Atr-I antibodies only identified epitopes in Atr-I, while the other two polyclonal antisera showed cross-reactivity.

**Table 3:** Epitopes discovered computationally (A), experimentally (B) and cross reactive regions (C).

Start	Sequence	End
**A Computationally predicted epitopes**		
**Atroxlysine-I**		**uniprot P85420**
4	QQR	6
11	FIVVDHGMF	19
27	DKIRRRIH	34
78	FGEWR	82
126	IQDHSEQDLM	135
152	HDTG	155
164	CIMS	167
179	SDCS	182
**Bap**		**uniprot P83512**
13	VVADHG	18
39	NTVGF	44
49	DVHA	52
77	KSFGEWRERD	86
161	GAKSCIMAS	169
176	SYEFSDCSQNQYE	188
**Leucurolysin**		**uniprot P84907**
11	VVADHGMFKKYN	24
38	NTVNGFFRSMN	48
78	FGEWRER	84
114	AGMCDLSQSVAVVMDHS	130
147	NLGMRHDGNQCHCNAPSCIMAD	168
176	FEFSDCSQ	183
**B Experimentally mapped epitopes**		
**Atroxlysine-I**		**uniprot P85420**
19	FMKYNGNSDKIRRRIHQMVNI	39
46	TMYIDILLTGVEIWSNKDLINVQPAAPQTL	75
**C Cross reactive regions**		
**Atroxlysine-I**		**uniprot P85420**
78	FGEWRKTDLLN	88
137	AITMAHELGHN	147
163	SCIMSPVL	167

### Receiver operating characteristic analysis shows an improvement in accuracy and a reduction in false positives

Three state-of-the-art prediction methods and our model were compared by the ROC curve analysis with default cutoffs that each software suggested. We also used cutoffs that maximized AUC and Precision (Table 4). We obtained mean AUC for the three proteins (Bap1, Leuc-a, and Atr-I) of 0.5407 followed by ABCpred (0.5382), TEPRF (0.5297), and BepiPred (0.4450). All the measurements showed that our predictor had better performance, accuracy, precision, true positive rate, and AUC while always having the lowest false positive rate (Table 4). The false positive rate was found to be significantly lower (0.3266) when compared to ABCpred (0.5752), BepiPred (0.3961), and TEPRF (0.5121).

**Table 4:** Comparison statistics of computational B-cell epitope prediction method.

Method	AUC	Accuracy	TPR	FPR	Precision	Specificity
**Default software cutoff statistics**						
ABCpred	0,5382	0,4384	0,6516	0,5752	0,0906	0,4248
Bepipred	0,4450	0,5655	0,2860	0,3961	0,1356	0,6039
Labimq	0,5407	**0,6175**	0,4080	**0,3266**	**0,2333**	**0,6734**
TEPRF	0,5297	0,4972	0,5714	0,5121	0,1097	0,4879
**Cutoffs that maximize precision**						
ABCpred	0,5525	0,5116	0,6275	0,5224	0,2041	0,4776
Bepipred	0,4825	0,5556	0,3976	0,4326	0,2117	0,5674
Labimq	0,5996	**0,7210**	0,4052	**0,2059**	**0,3157**	**0,7941**
TEPRF	0,5878	0,3790	0,9896	0,8140	0,2407	0,1860
**Cutoffs that maximize Area Under the Curve**						
ABCpred	0,6542	0,6722	0,6617	0,3534	0,1255	0,6466
Bepipred	0,5499	0,6650	0,3799	0,2802	0,1443	0,7198
Labimq	0,6306	**0,7876**	0,4189	**0,1577**	**0,2852**	**0,8423**
TEPRF	0,6330	0,4912	0,8464	0,5805	0,1742	0,4195

### Experimental validation of epitope prediction by immunization of mice

We selected two Atr-I regions to be synthesized as peptides by Fmoc chemistry for antibody production. One epitope corresponding to the N-terminal region (9VDLFIVVDHGMFMKY23) was identified by our model with a prediction score of 0.49; we also selected a central region (99LTSTDFNGPTIGLAY113), which was not identified by our model. Both regions were not mapped in SPOT experiments. We chose a cutoff of 0.2 to classify a sequence as positive. The peptides were called AtrCPEN (Computationally Positive Experimentally Negative) and AtrCNEN (Computationally Negative and Experimentally Negative), respectively. Their molecular masses were verified by mass spectrometry after synthesis and corresponded to the predicted amidated and acetylated masses (not shown).

**Figure 3:**
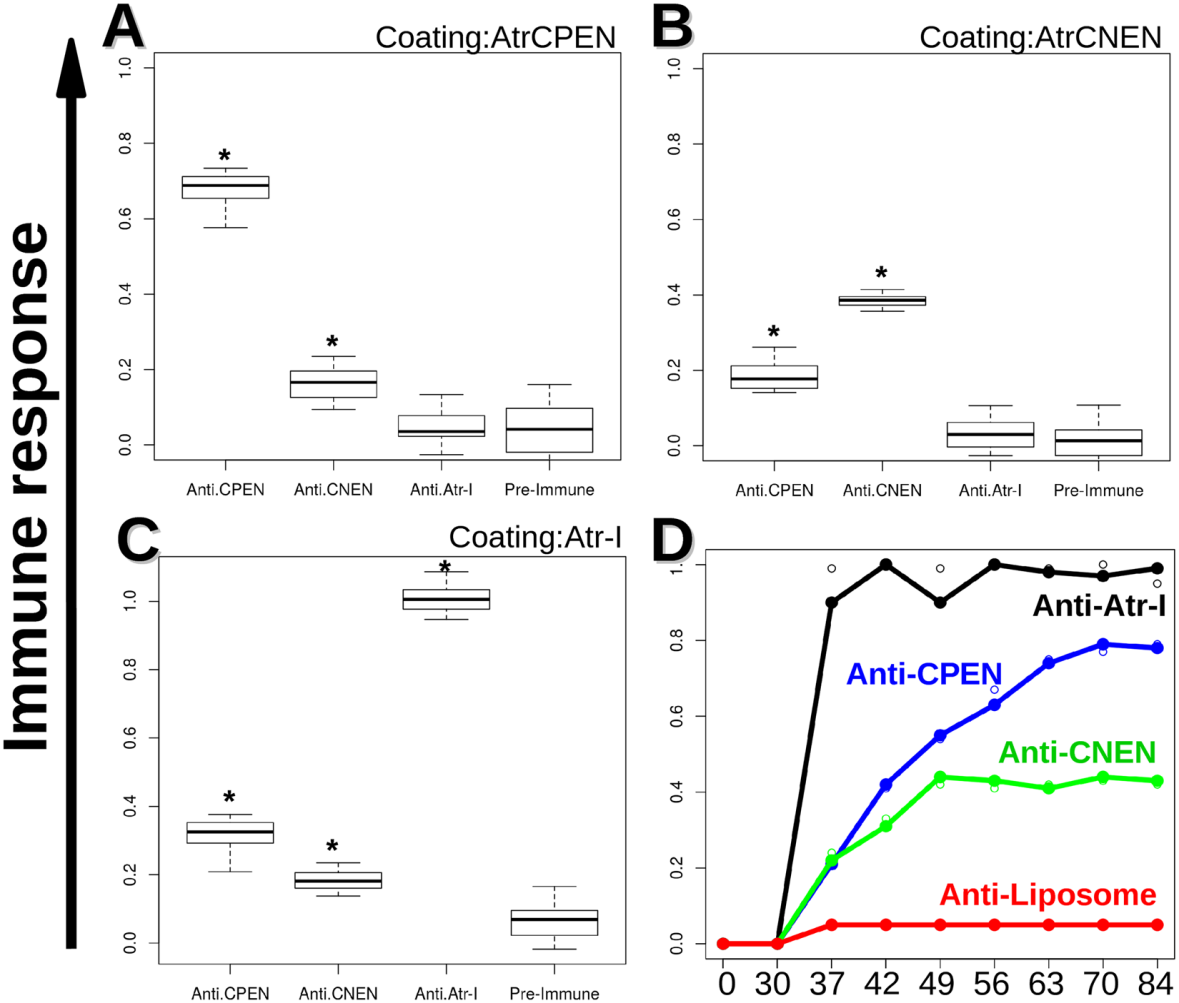
ELISA tests of anti-CPEN, CNEN, and Atr-I sera. (**A**–**C**) show the boxplots results for ELISA plates coated individually by CPEN, CNEN, and Atr-I, respectively. |Plates were incubated for 1 h with the respective sera at 37 °C followed by another round of 3× washing before incubation with a respective secondary antibody for 1 h. An OPD substrate was added for ~20 minute incubation, and the reaction was stopped with H_2_SO_4_ prior plate reading. The black lines within the boxes correspond to medians. All the samples marked with (*) show to be significantly different for a p < 0.05, when comparing by the *t* test available in software R. (**D**) shows antibody binding over 9 doses (x-axis represents days).

AtrCPEN and AtrCNEN peptides were utilized for immunization of BALB/c mice, after incorporation into liposomes as an adjuvant. An ELISA was conducted to monitor antibody production against AtrCPEN and AtrCNEN. It was possible to detect specific antibody production after the 7th dose (day 63; Fig. 3D). The two synthesized peptides were not recognized by anti-Atr-I serum. By contrast, anti-AtrCPEN (0.3 Abs.) recognition of Atr-I as an antigen was slightly higher than that obtained by Anti-AtrCNEN (0.2 Abs; Fig. 3B). Antipeptide sera (Anti-AtrCPEN and Anti-AtrCNEN) only poorly recognized Atr-I. The antibody responses were compared by the *t* test showing a p-value lower than 0.05 for all groups (confidence interval: 95%).

To verify whether the produced antibodies against Atr-CPEN and Atr-CNEN had neutralizing properties against Atr-I, we tested the enzymatic activity of Atr-1 over time, in the presence of anti-CPEN and anti-CNEN, using a synthetic substrate, Abz-LVEALYQ, that produces fluorescence when cleaved (Supplementary File 3). This assay showed Atr-I activity reduction by 80–70% for anti-CPEN and 30–20% for anti-CNEN. This result indicated the successful neutralization of Atr-I activity, when incubated with anti-CPEN; this neutralization was significantly stronger than that obtained with the anti-CNEN antibody.

**Figure 4:**
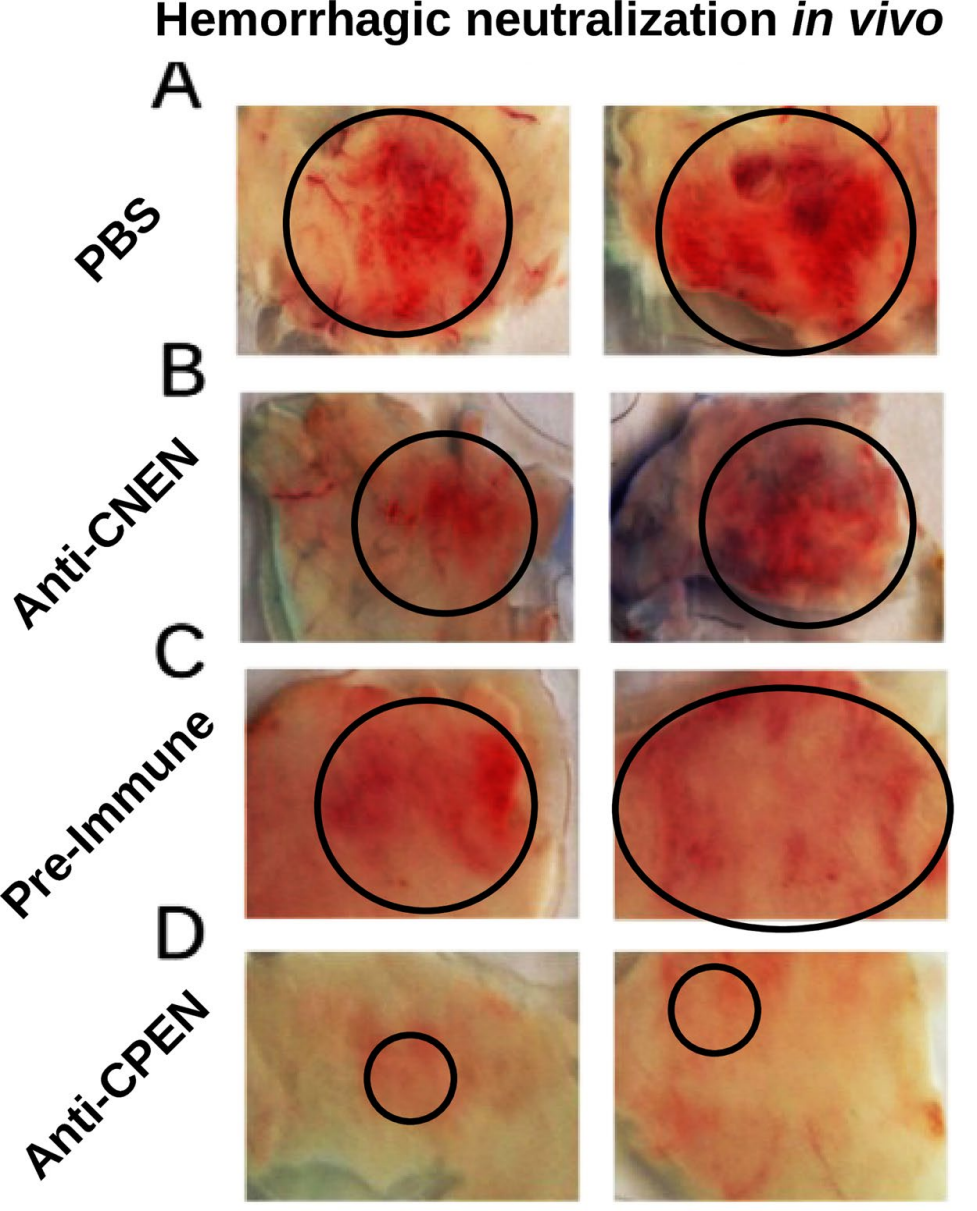
Hemorrhagic Atr-I activity neutralization *in vivo*. Mice were challenged with 1 MHD (Minimal Hemorrhage Dose) of Atr-I diluted either in PBS (**A**) anti-CNEN serum (**B**) preimmune serum (4C) or anti-CPEN serum (**D**). Black circles indicate hemorrhagic areas in animal skin, for each treatment. (**A**–**C**) showed a clear hemorrhage area, while serum against CPEN (shown in **D**) was able to reduce hemorrhage, causing only skin irritation.

Because Atr-I activity neutralization by anti-CPEN antibodies was observed *in vitro*, Atr-I-induced hemorrhage neutralization was tested *in vivo*, in BALB/c mice. They were challenged with a toxin amount corresponding to 1 Minimum Hemorrhage Dose (19 μg of Atr-I in 100 μL), as described by Schneider *et al*., 2016. The animals challenged with Atr-I mixed with anti-AtrCPEN serum showed a clear reduction in hemorrhage when compared to the positive control group. Negative control with preimmune serum samples and anti-AtrCNEN serum yielded slightly reduced hemorrhage when compared with the positive control, probably owing to other serum components that interfere with the enzymatic metalloendopeptidase activity (Fig. 4).

**Figure 5:**
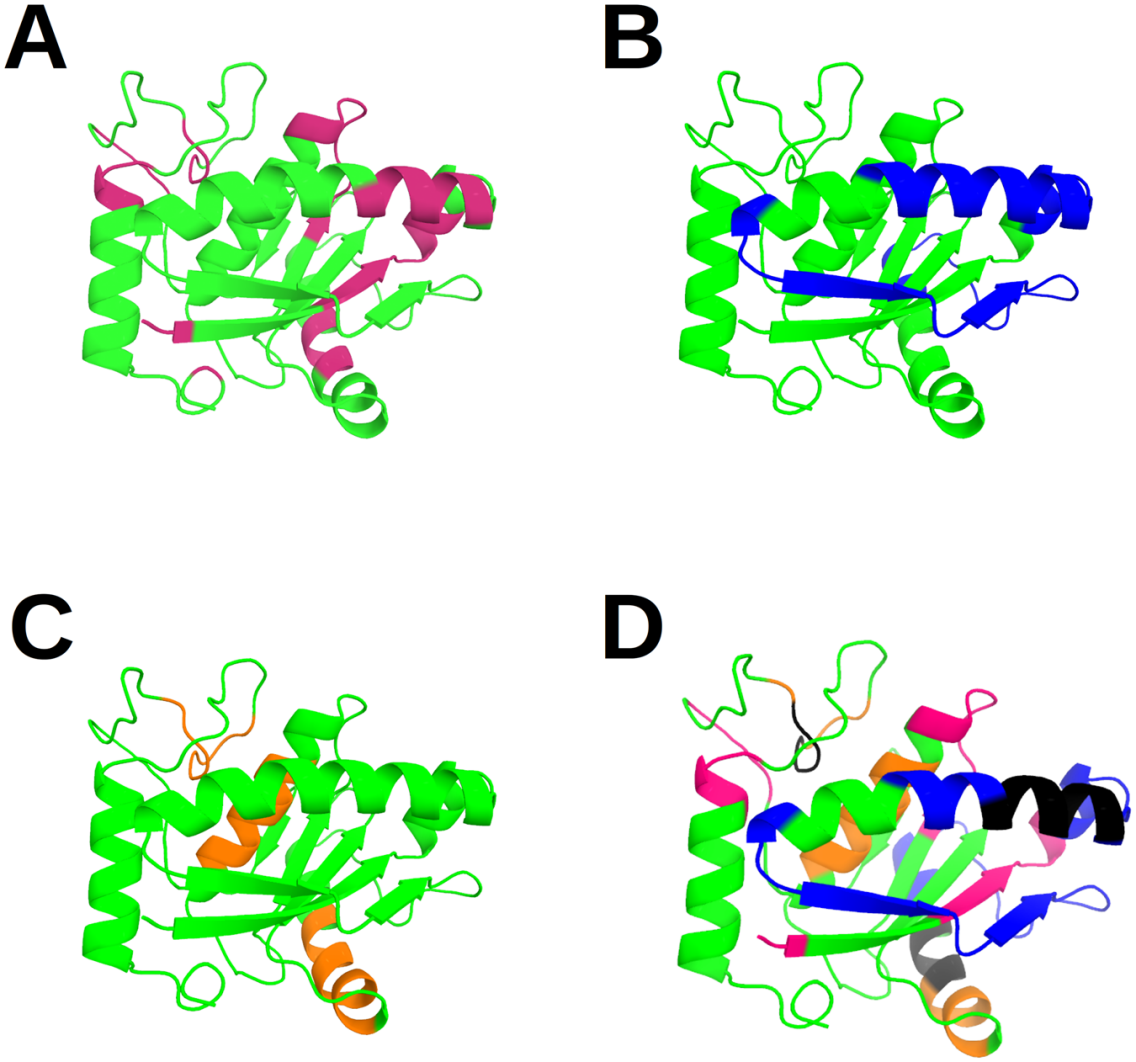
Localization of predicted epitopes in the Atr-I model. A cartoon view of the structural model of the protein Atr-I is displayed. The residues that belong to the computational prediction are shown in pink (**A**) while the experimental epitopes and the cross-reactive regions between the different serum samples tested are indicated in blue (anti-Atr1) and orange respectively (anti-Bap1 and anti-Leuc-a) (**B**,**C**). The overlap of these methods is presented in (**D**) where the black regions correspond to the matching computational and experimental predictions.

Spatial distribution of epitopes obtained by computational and experimental methods showed an overlapping region, whereas cross-reactive regions did not seem to have any pattern (Fig. 5).

## Discussion

B-cell epitopes are related to a humoral immune response and play a key role in vaccine production and several biotechnological applications, while T-cell epitopes are associated with cell-mediated immunity^29,30^. The experimental epitope identification is time- and resource-consuming in comparison with other computational techniques^2^. Computer algorithms to predict B-epitopes by means of an antigen sequence^3,4^ or structure^9,13,31^ have been refined over the past decades. These techniques have been accompanied by experimentally characterized datasets comprising both positive epitopes and negative nonepitopes^15^.

The first challenge for epitope prediction is represented by database construction aimed at organizing the disproportional negative or nonepitope examples^16^. Another complication is the negative example selection, based on randomly chosen sequences, where no antibody binding is reported^22^. Collections of both epitopes and nonepitope sequences from experimentally validated data are available, e.g., the Immune Epitope Database (IEDB)^16^. Nevertheless, experimental nonepitope data still have the potential for being due to possibly flawed result interpretation, a lack of detailed mapping, or simple experimental errors^21^. Furthermore, this principle applies to all existent experimental and computational methods as demonstrated in our results dealing with the production of neutralizing antibodies having a region undetected by experimental mapping.

The factors that influence the immune response and epitope detection are mostly attributed to genetics^19^, evolution^9^, immunological complexity^5,18,20^, structural conformation^32^, surface indistinguishability^15^, and others^31^. Moreover, we recently explored the differences between epitopes from different antigen classes or families and revealed that these differences can be useful for identification of epitopes^23^. We took advantage of the antigen sequence from a single protein class (metalloproteinases) to train a decision tree classifier. Furthermore, we validated our hypothesis by comparing our results to those of state-of-the-art predictors and experimental methods for three different proteins, thus showing an accuracy improvement and a reduction in the false positive rate (Table 4). The important attributes for classification included the isoelectric point, lateral chain size, and amino acid residues such as Asn, Gln, Ser, Thr, Lys, or Trp (Table 1), which are described as antigenic^2,6,7^. Other studies indicate that sequence attributes can be used for analyzing structural^33^ and sequential epitopes^34,35^, thus highlighting hydrophilic amino acids because they surround antigenic determinants^36^.

Classification models are great tools for identifying patterns within complex data and gradually gain importance in computational biology owing to rising information amounts^37^. Machine-learning approaches have undisputed advantages over simpler methods, such as regression^38^, but their persistent limitations are the inability to point out relevant characteristics and the necessity of high computational power^34^. Regression-based methods such as random forest^39^, decision tree^40^, and linear regression^11^ allow researchers to identify these attributes rapidly^41,42^ (Table 1, Supplementary File 2).

Here, we show an improvement in the performance on *in silico* epitope prediction (Table 4) and *in vivo* validation (Figs 2 and 4), especially in terms of the false positive rate, when compared to other methods (BepiPred, ABCpred, and TEPRF). BepiPred employs hidden Markov models to identify propensities in sequence data, despite underperformance of similar approaches, as uncovered by Blythe and Flower^6,32^. ABCpred is a method that involves recurrent neural networks to analyze fixed length windows of less than 20 amino acids and their biochemical properties^43^. The random forest approach (TEPRF) takes advantage of two powerful machine-learning techniques: bagging (bootstrap) and random attribute selection. TEPRF yields a large number of false positives that could be explained by the attempt at overpredicting the under-represented groups or experimental epitopes. To overcome these limitations, we increased the negative/positive examples for epitopes by separating the full sequence into Kmers of several lengths (3 to 15 aa) and by correcting the proportions with SMOTE, thus increasing overall performance (Table 4).

A major point poorly discussed in the literature is that experimental nonepitopes are classified as false positives and are employed as such by predictors^30^. We demonstrated that one of these epitopes predicted and experimentally mapped as a negative epitope (CPEN) was able to induce neutralizing antibodies (Figs 3 and 4). This finding also means that statistical comparisons for the current classification methods harbor a bias that significantly alters the accuracy of current predictors. This bias could be due to variation in immunological or experimental conditions. Continuous epitopes are powerful diagnostic/treatment tools, despite representing only 10% of all estimated B-cell epitopes^14,44,45^.

The low accuracy seen during prediction validation across the different algorithms could be due to the statistical bias caused by incomplete experimental results or database failure. Most amino acids identified as epitopes *in silico* by us were in the proximity of (or were partially included in) experimental epitopes (Fig. 2). This is important because linear epitope sequences can be rapidly produced as soluble peptides for immunization^1,29^. These peptides are flexible^34^ and have a higher probability of mimicking epitopes^46^. Peptides designed on the basis of these epitopes are undoubtedly powerful tools for improving vaccine efficacy^38,47^.

This study was focused on the snake venom metalloendopeptidase called Atroxlysin-I as a candidate protein for our experiments because it is a well-characterized enzyme. It can enzymatically cleave Xaa-Leu bonds in proteins such as fibrin, fibronectin, type I and IV collagens, and other extracellular-matrix components and can induce hemorrhage. Besides, Atr-I interferes with platelet aggregation in an enzymatically independent manner^1^.

Of note, in this study, it was shown that a predicted epitope for Atr-I (AtrCPEN), not identified by experimental mapping, can be employed to induce neutralizing antibodies. The region identified (aa 11–19) was expanded to aa 8–22 for synthesis purposes; this approach non significantly reduced the score of our model from 0.6 to 0.5, whereas any sequence with a score above 0.2 was regarded as an epitope (Fig. 2). The sequence chosen to represent a negative prediction had a score of zero, and it was not recognized by experimental procedures. Both peptides induced antibody production with a lower response to Atr-I than its corresponding anti-Atr-I sera (Fig. 3). The lower response associated with peptide-based antibodies has been observed previously^14^. Atr-I was experimentally found to contain two immunogenic regions in the regions aa 19–39 and aa 46–75, as identified with anti-Atr-I serum obtained elsewhere^48^. Our prediction match region (positions 27–34) is similar to BepiPred’s (positions 23 to 29) (data not shown), which are closer to the border of the experimentally identified region^48^. TEPRF and ABCpred select larger protein portions, reflected in a higher false positive rate (Table 4). The experimental epitopes found in Leuc-a, Bap1, and Atr-I contain a common region between residues 28 and 39 (Atr-I), while the two peaks for Atr-I: one, for Bap-1, and the other for Leuc-a, are encapsulated between positions 19 and 64.

The Atr-I structural model revealed that anti-Atr-I sera recognize two regions near the N-terminal portion of Atr-I (Fig. 5). All three analyzed endopeptidases had epitopes located in the region comprised by the first and second sequence portion close to the N-terminal amino acid, as shown by orange, blue, and green color peaks (Fig. 2). This region comprises two α-helices with a loop in between, followed by another loop and a strand. These helix-loop regions were shown to be immunoreactive with anti-Atr-I sera and with cross-reactive sera as well (Fig. 5). The preference of anti-Bap1 sera matched our predictions as well on the first helix-loop with residues 19–39. This structure seems to be conserved among all metalloendopeptidases and could be a source for the development of additional antivenom agents and vaccines against other toxic endopeptidases. This region was erroneously labeled as an experimentally negative region, but we presented evidence to the contrary, and we were able to neutralize the hemorrhagic effect of Atr-I (Fig. 4). The impact of this approach during epitope predictions is clear, and some regions erroneously classified by experimental methods can harbor immunogenic properties (Fig. 2). The other two regions next to aa 65–75 may point to another region that was immunodominant for anti-Leuc-a sera whereas anti-Bap1 showed a preference for a region close to the C-terminal Atr-I segment. These untested regions may be important for other endopeptidases owing to their cross-reactivity and structural identity.

An immunogenic region depends on the system where it is identified and on the parameters of this system^49^, such as the immunized host type, antigen type, inoculation method, adjuvant presence, and others^50^. These epitopes can be refined for vaccination^51^ and their biotechnological applications are well known^1,29^. Several computational methods are available^32^, and their results are in agreement within consequences of certain conditions, in which amino acids, hydropathy values, and others are relevant for immunodominance^49^. These properties escalate to form complex networks and energetic mechanics^52^ thus making the task of predicting B-cell epitopes a major challenge^32^. Computational and experimental methods suggest that regions helix-loop, sheet-loop, helix-loop-helix, and helix-loop-sheet are the most likely to result in epitopes, binding, and therefore detection and neutralization of a target protein^53^. Besides, this study showed an improvement in epitope prediction accuracy by revealing a specific approach to the still complicated task of predicting neutralizing epitopes and vaccine targets.

## Conclusion

This work describes development of a classification model based on a protein dataset that belongs to a single antigen class (metalloendopeptidases), but the method can be applied to any protein class. This model successfully predicted linear epitopes that overlap with experimentally determined epitopes on three sample proteins, with better performance than ABCpred, BepiPred, and TEPRF. We also determined which biochemical attributes are important during epitope prediction for this model. Furthermore, antisera raised against these epitope regions were demonstrated to be cross-reactive and will improve the understanding of the immunoreactive regions in metalloendopeptidases. Furthermore, we produced neutralizing antibodies against Atroxlysin-I through immunization with a synthetic peptide. The selected region was based on a predicted positive but experimentally negative epitope. Therefore, it was demonstrated here that computationally positive predictions can serve as a basis for producing peptides capable of raising neutralizing antibodies.

## Methods

### Ethics statement

The study protocol was approved by the Ethics Committee for Animal Experimentation, Universidade Federal de Minas Gerais (protocol number 200/2010). All the experiments were performed in accordance with Guide for the Care and Use of Laboratory Animals, US National Institutes of Health (NIH Publication No. 85-23, revised 1996).

### Animals and venoms

The animals were maintained at Centro de Bioterismo and received water and food under controlled environmental conditions (Instituto de Ciências Biológicas, Universidade Federal de Minas Gerais, Brazil). A venom pool from at least six adult Peruvian *B. atrox* specimens was donated by the Instituto Nacional de Salud (Lima, Peru). Purified Atr-I was previously obtained in our laboratory, as previously described^1,9^, ICB-UFMG.

### Dataset

The B-cell epitope dataset (Rong and Jianjun, 2011), was used and modified, as previously by us elsewhere^23^. Briefly, this dataset contains manually curated and selected metalloendopeptidases based on the experimental validation procedures as described (Supplementary Data 1).

### Attribute matrix analysis

We produced a matrix based on the properties of Kmers between 3 and 15 aa derived from every protein and epitope in our dataset. The Kmer sizes were chosen according to a common epitope size distribution^54^. The attributes are the percentages of each of the 20 aa; sequence lengths; hydropathy index; atom percentages of C, H, O, N, and S; the aliphatic chain size index; isoelectric point; and amino acid percentages grouped by hydrophobic, positive, negative, polar, and special amino acids (CGP).

The Kmers were tagged as an epitope or nonepitope if they had 50% or more aa that belonged to the respective class. The attributes were computed by a Biopython package for Python^55^ and Perl scripts based on Expasy descriptions (Wilkins MR., *et al*. (1999)). We selected a decision tree model as a data-mining technique for classifying the Kmers according to their attributes. We also chose the ClustalO^56^ local alignment option for aligning the metalloendopeptidases Bap1 (*B. asper*), Leuc-a (*B. leucurus*), and Atr-I (*B. atrox*).

### Classification of computational epitopes

The decision tree classifier tends to overpredict the majority class. To avoid this bias, the positive and negative sequence proportion was altered by means of the SMOTE algorithm^57^ for over-representing the minority class and improving the performance of decision tree models.

A classification model series was tested using KNIME^58^. The decision tree was selected as the most suitable method because of speed and performance^23^. The important attributes were compared with two measures (decreased Gini and accuracy) produced by randomForest Package on R, after a million trees produced by means of the same attributes as in our decision tree model. These measures are defined as inequity and inclusion measures, respectively.

### Statistical validation

Statistical analysis was carried out in R^59^, as described elsewhere^23^ and included 10-fold cross-validation (Krstajic., *et al*. (2003)). This classification model performance was studied by analyzing a receiver operating characteristic (ROC) curve^17^, recall, precision, specificity, area under the curve (AUC), and Cohen’s Kappa coefficient^39^. Mean comparisons between experimental and computational results were conducted by the *t* test available in the R software.

### Cellulose-bound peptide production and immunoassay

Briefly, A Multipep (Intavis) robot was used for automating peptide synthesis of overlapping pentadecapeptides frame-shifted by 3 residues covering the entire amino acid sequence of Atr-I, Leuc-a, and Bap1 on cellulose membranes. Later, these membranes were tested against specific serum samples, as previously described by us^26,27,48^.

### Soluble peptide synthesis

Fmoc amino acids were acquired from Novabiochem or Sigma Aldrich. After epitope prediction, two linear regions were selected for synthesis. The first region was localized near the N-terminal region of Atr-I (11-FIVVDHGMF-19). We increased the predicted sequence by 3 aa for each border, resulting in a final sequence of 15 aa with composition 9-VDLFIVVDHGMFMKY-23 (AtrCPEN). A second region is localized in the central part of the protein 99-LTSTDFNGPTIGLAY-113 (AtrCNEN). AtrCNEN was also 15 aa long and showed no previous immunological response and was undetectable by the computational methods tested in this study. The two peptides were synthesized by the Fmoc chemistry method on an automatic Multiprep robot (Intavis)^60^. During the synthesis, the growing peptide was immobilized on the Rink Amide resin (Novabiochem). At the end of the synthesis, peptides were released from the resin, and the side chain deprotection was carried out by trifluoroacetic acid treatment (95% TFA, 2.5% triisopropylsilane, and 2.5% water). The molecular masses of synthesis products were analyzed by mass spectrometry (MALDI-TOF, linear mode).

### Production antipeptide sera

Two groups of five BALB/c female mice were immunized subcutaneously with AtrCNEN or AtrCPEN. Each peptide was encapsulated into Asolectin liposomes (Sigma Aldrich), as described by us elsewhere^27^. Aluminum hydroxide (40 μg/μL) was added in a 1:1 (v:w) ratio as an adjuvant. All animal groups received 10 doses during a 3-month protocol with the initial 1-month interval, followed by weekly doses. Experimental groups received 50 μg of an encapsulated peptide per dose per animal. Control animals were immunized with empty liposomes (without an antigen) in aluminum hydroxide (40 µg/µL). One week after the last immunization, the mice were bled to recover the immunized serum.

### Antigenic anti-AtrCNEN and anti-AtrCPEN analysis by ELISA

Recognition of the synthesized peptides by BALB/c IgG anti-Atr-I antibody (previously produced and kindly donated by Sanchez *et al*.^26^) was tested in Maxisorp plates (Nunc) coated with AtrCNEN, AtrCPEN, (10 μg/mL), or Atr-I (5 μg/mL) overnight at 4 °C in coating buffer (0.05 M Na_2_CO_3_, pH 9.6). After blockage for 1 h at 37 °C with a powdered milk solution (2%) in PBS containing Tween 20 (0.1%), IgG Anti-Atr-I, AntiAtrCPEN, or AntiAtrCNEN produced in BALB/c mice was incubated for 1 hour at 37 °C. A goat anti-mouse IgG antibody conjugated to peroxidase (Sigma) served for detecting the reaction followed by addition of the OPD Peroxidase substrate (SIGMAFAST from Sigma-Aldrich).

### Methods for computational evaluation and comparison

The immunoassay data from cellulose-bound peptides were employed for calculating a reactivity score for the three out-sample proteins uncharacterized at the time (Bap1, Leuc-a, and Atr-I). The score is based on the aa occurrence numbers in each predicted overlapping reactive peptide and later was scaled to a maximum value of 1. These results were compared with our model, BepiPred, ABCpred, and TEPRF^7,43^.

### Experimental validation

The Atr-I protein and sera against Atr-I Leuc-a and Bap1 were used to experimentally validate the classification model. We selected one region from Atr-I, considered immunogenic only by our predictor and a second region considered immunologically negative by all the tested mapping methods (i.e., computational and experimental). These two peptides were synthesized and used for producing antibodies and were later characterized. In addition, we compared the cross-reactivity of anti-Atr-I, anti-Bap1, and anti-Leuc-a sera (Kindly donated by Schneider, F. *et al*.^61^ against each sample protein. Immunoassays with cellulose-bound peptides were conducted. Besides, we compared these results to the predictions. Finally, all the comparisons between experimental and computational methods were expressed in accuracy, precision, recall, AUC, and ROC curves. Furthermore, we compared the epitope spatial distributions using a 3D model built with a combination of methods^35,62,63^.

### An *in vitro* neutralization assay

The FRET peptide (Abz-LVEALYQ-EDDnp) kindly donated by Schneider *et al*.^61^. was used to test the neutralizing activities of antiCPEN and antiCNEN after Abz-LVEALYQ-EDDnp hydrolysis by purified Atr-I also donated by Schenider *et al*.^61^, 2016. First, 11 ng of Atr-I was preincubated with 1, 2, or 3 µg of antiCPEN or antiCNEN for 30 min at 37 °C. Then, the substrate was added at a final concentration of 47 mM. Positive controls were set up by preincubating Atr-I alone for 30 min at 37 °C. The residual activity and neutralizing activity were normalized to the positive control. Enzymatic activity was measured by fluorescence on a Sinergy2 (Biotek) instrument (λ_ex_ = 320 nm and λ_em_ = 420 nm) for 30 min at 37 °C as described by Schneider *et al*.^61^.

### An *in vivo* neutralizing assay

Atr-I–induced hemorrhage neutralization was tested in 16 BALB\c mice separated into groups of four. One minimum hemorrhagic dose (MHD/kg) of Atr-I (19 μg per mouse of 18–22 g)^48^ was pre-incubated with 50 μL of either anti-AtrCNEN or anti-AtrCPEN sera for 1 hour at 37 °C. The mixtures were inoculated subcutaneously into four mice per group. As a positive control, Atr-I was injected alone (without antisera). The negative control involved preimmune sera. After 3 hours, the mice were euthanized and their skin was removed for evaluating hemorrhage.

## Electronic supplementary material

Proteins used as training for the decision tree

Supplementary figures

## Data Availability

All the data depicted in the figures are available upon request.
