# Supplementary material for: Computational B-cell epitope identification and production of neutralizing murine antibodies against Atroxlysin-I
Source: Sci Rep. 2018 Oct 8;8:14904. doi: 10.1038/s41598-018-33298-x (PMC6175905; doi:10.1038/s41598-018-33298-x)
Supplement: Supplementary file 2 — Supplementary figures [file 41598_2018_33298_MOESM2_ESM.pdf]

Tittle: Computational B-cell epitope identification and production of neutralizing murine antibodies against Atroxlysin-I

Authors: *Edgar Ernesto Gonzalez Kozlova1, Loïc Cerf2, Francisco Santos Schneider3, Benjamin Thomas Viart4, Christophe NGuyen3, Bethina Trevisol Steiner5, Sabrina de Almeida Lima1, Franck Molina3, Clara Guerra Duarte1, Liza Felicori1, Carlos Chávez-Olórtegui1 and Ricardo Andrez Machado-de-Ávila5

Figure tittle: Decision tree for classification of epitopes

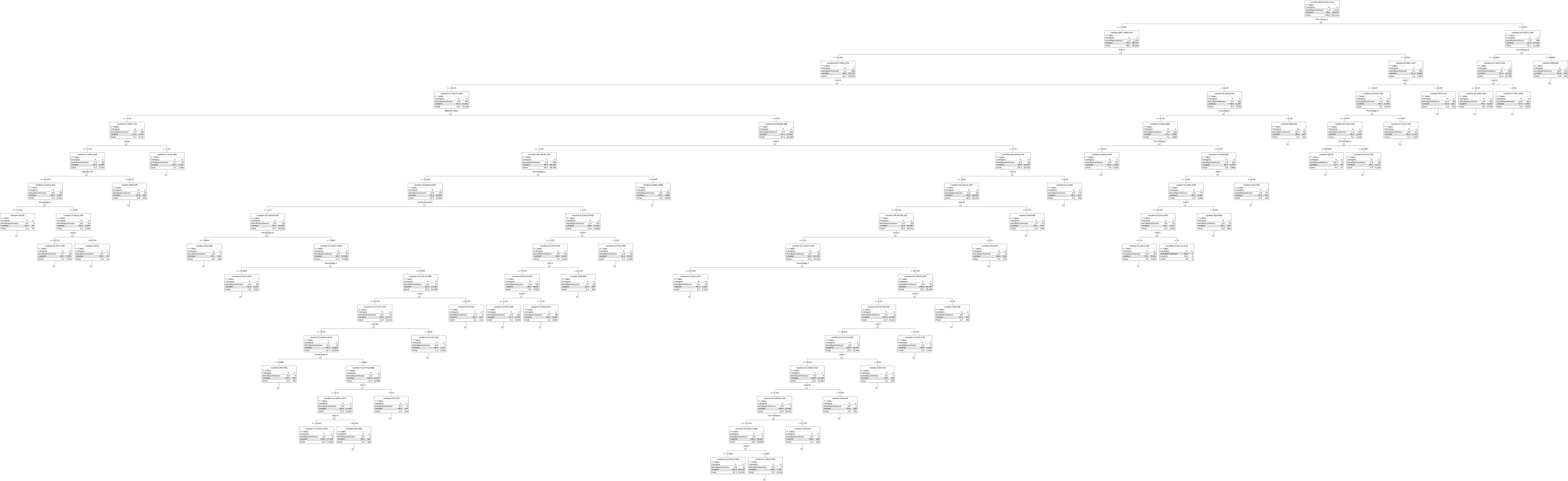

A

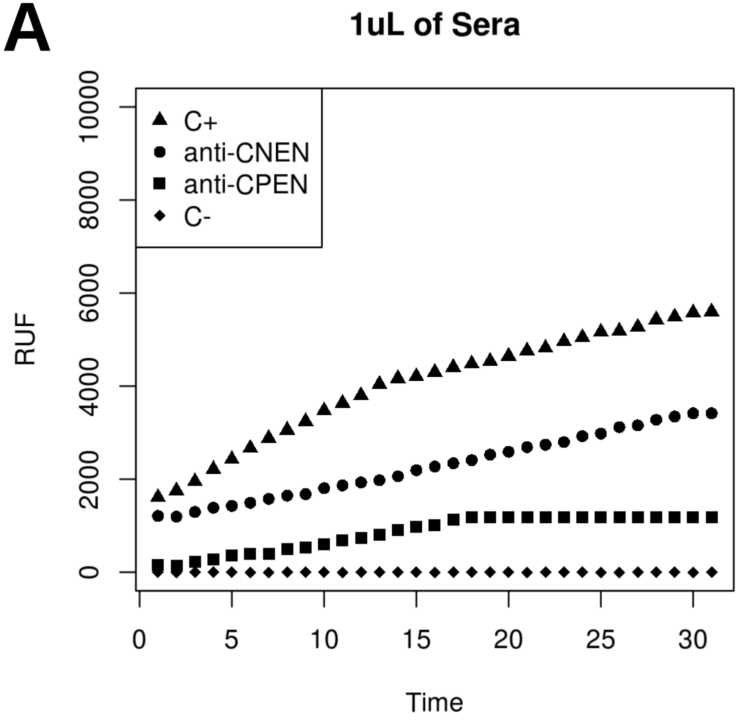

C

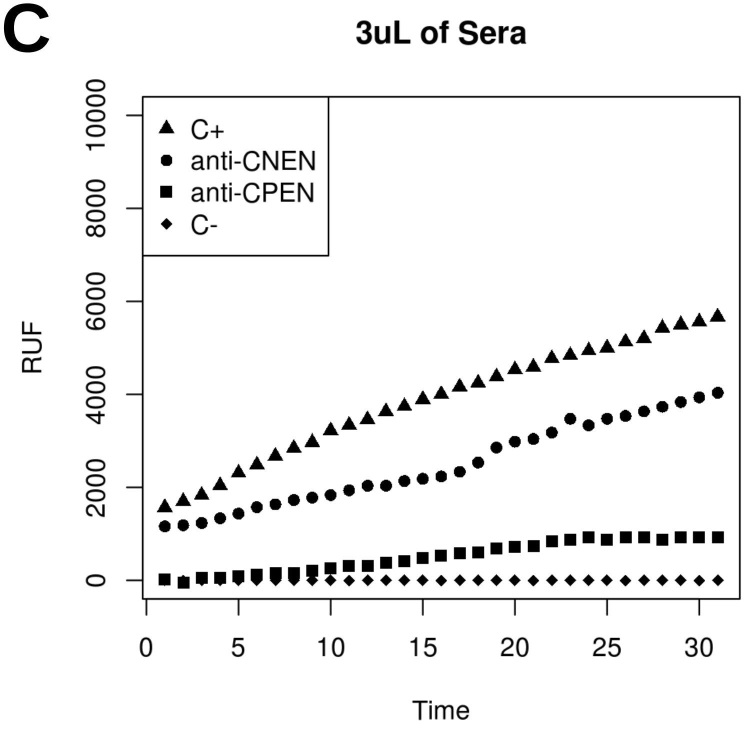

B

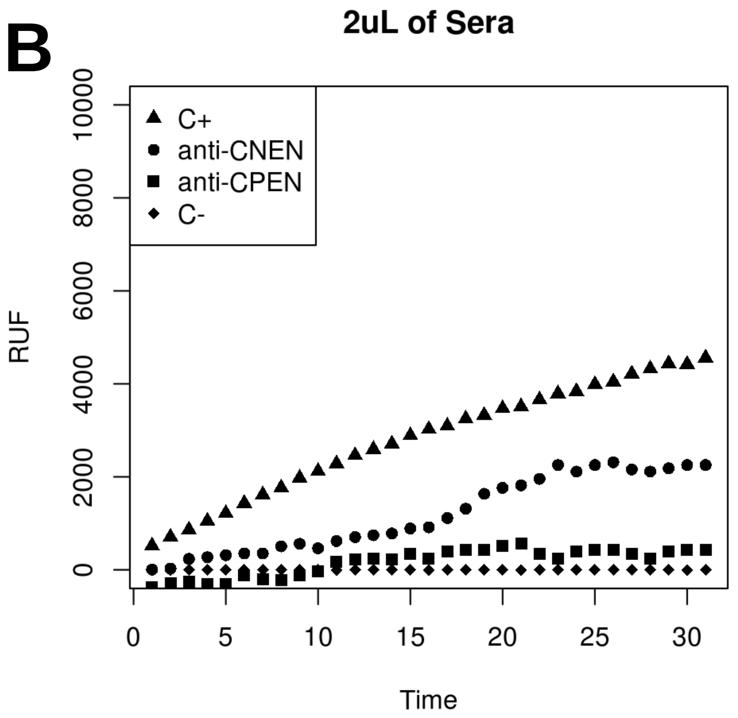

Computational B-cell epitope identification and production of neutralizing murine antibodies against Atroxlysin-I

*Edgar Ernesto Gonzalez Kozlova1, Loïc Cerf2, Francisco Santos Schneider3, Benjamin Thomas Viart4, Christophe NGuyen3, Bethina Trevisol Steiner5, Sabrina de Almeida Lima1, Franck Molina3, Clara Guerra Duarte1, Liza Felicori1, Carlos Chávez-Olórtegui1 and Ricardo Andrez Machado-de-Ávila5

Supplemenary file 3. Enzymatic hydrolysis of Abz-LVEALYQ-EDDnp by Atr-I. Cleavage of Abz-LVEALYQ-EDDnp caused by Atr-I results in a fluorescent emission dependent of the Atr-I enzymatic activity. The hydrolysis is measured over 30 minutes for 4 samples, simultaneously. A positive control of hydrolysis (Atr-I); The Atr-I in presence of serum anti-CNEN, anti-CPEN and a negative control with theAbz-LVEALYQ-EDDnp alone. The figures A, B, C show the enzymatic activity for 3 different quantities of anti-sera used. 1UL, 2uL and 3uL, respectively.
